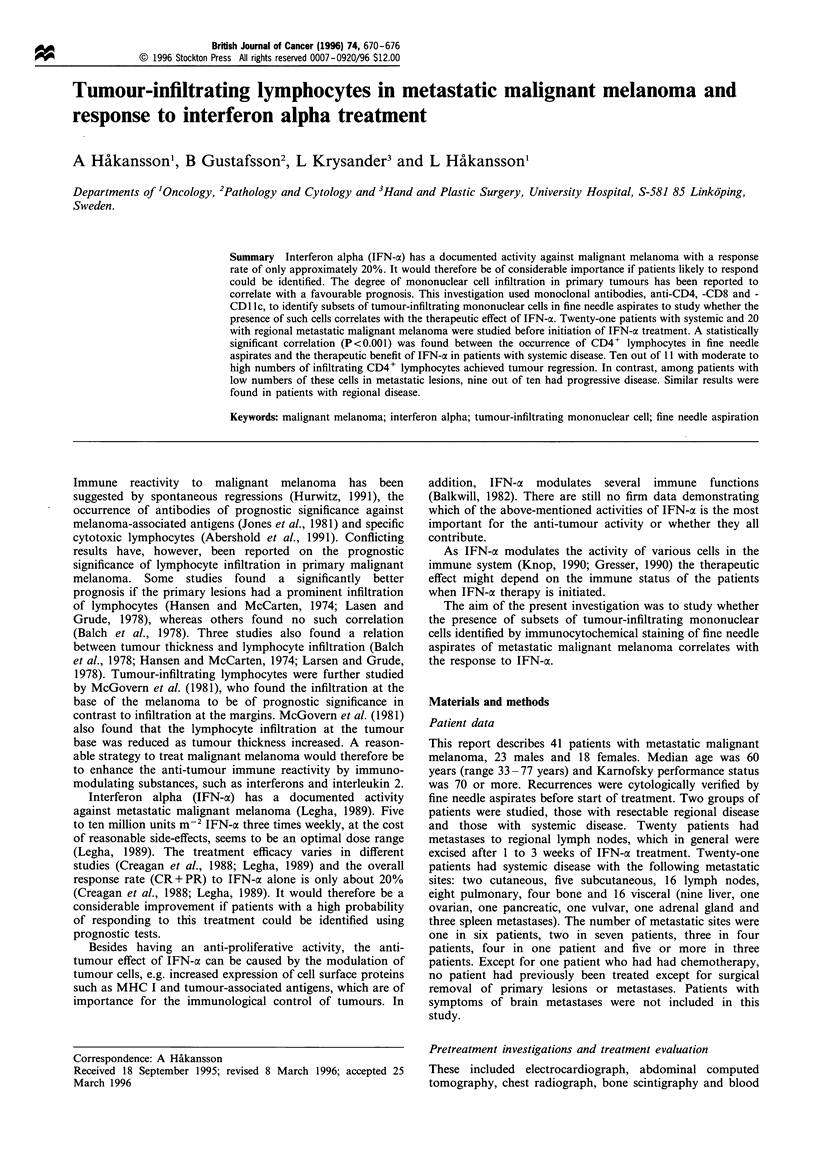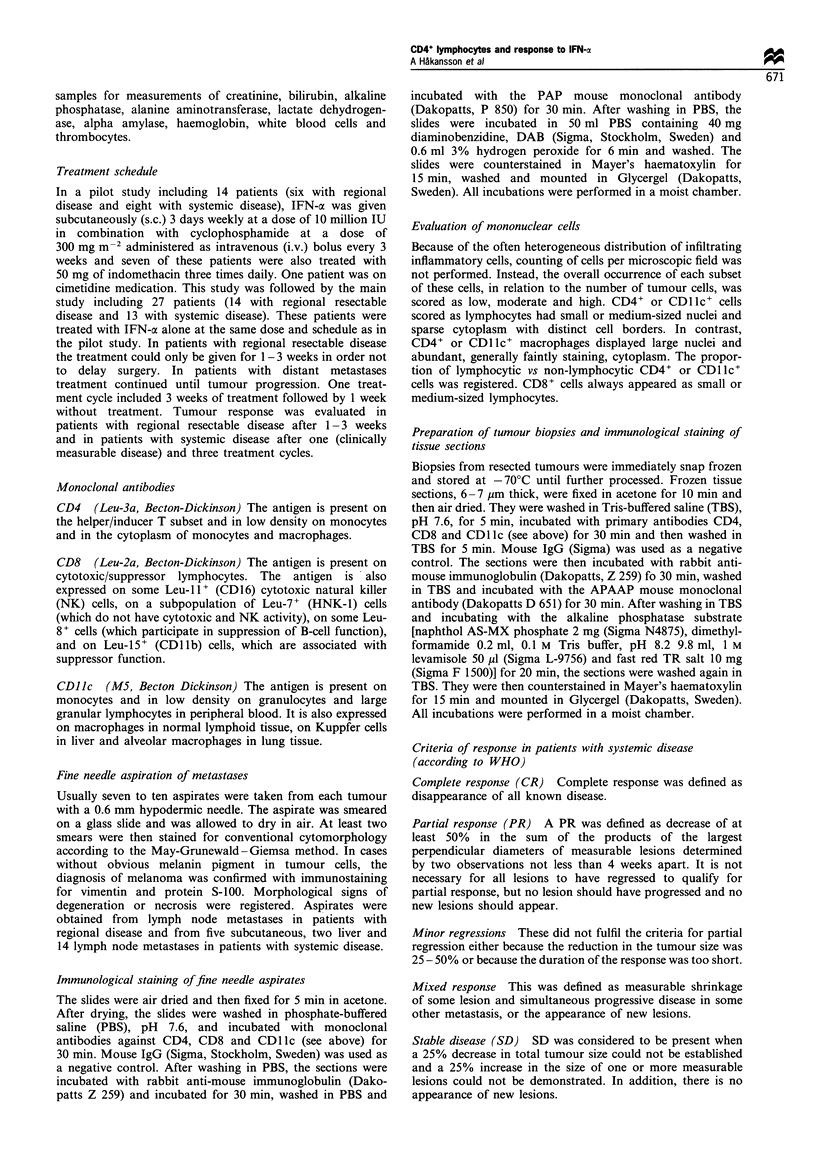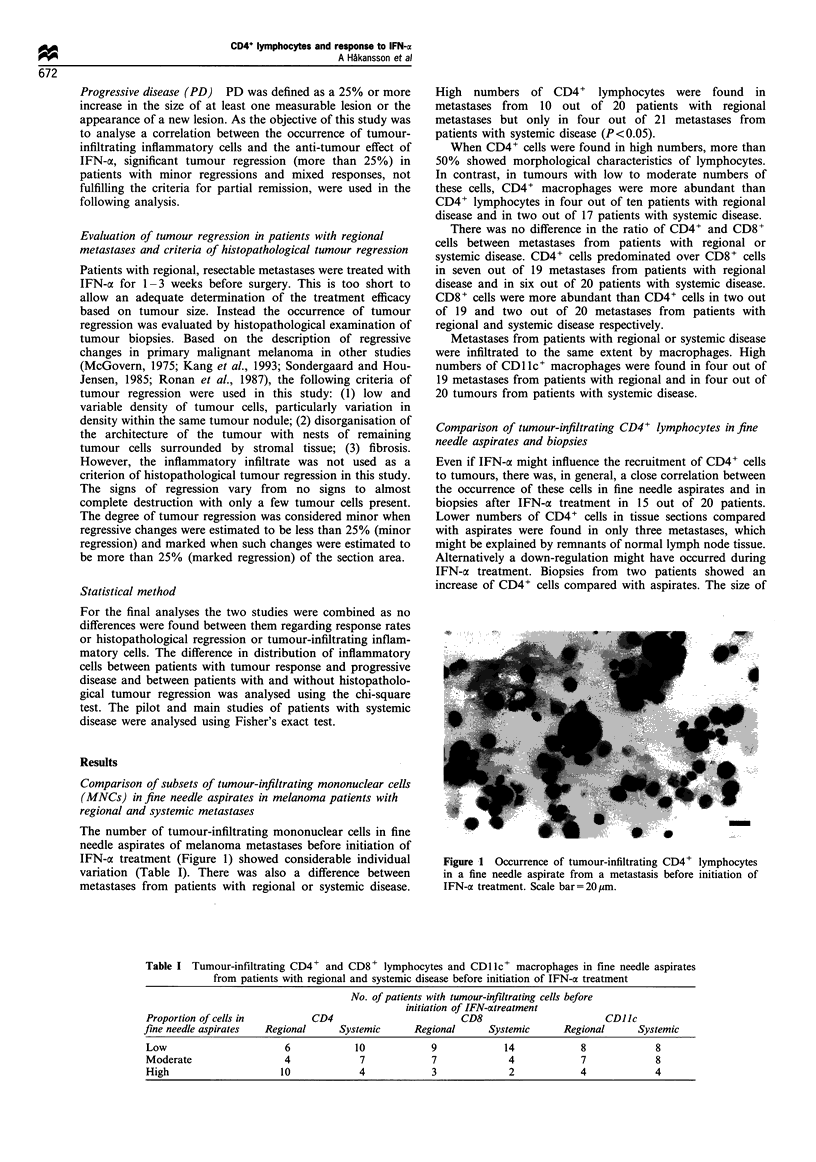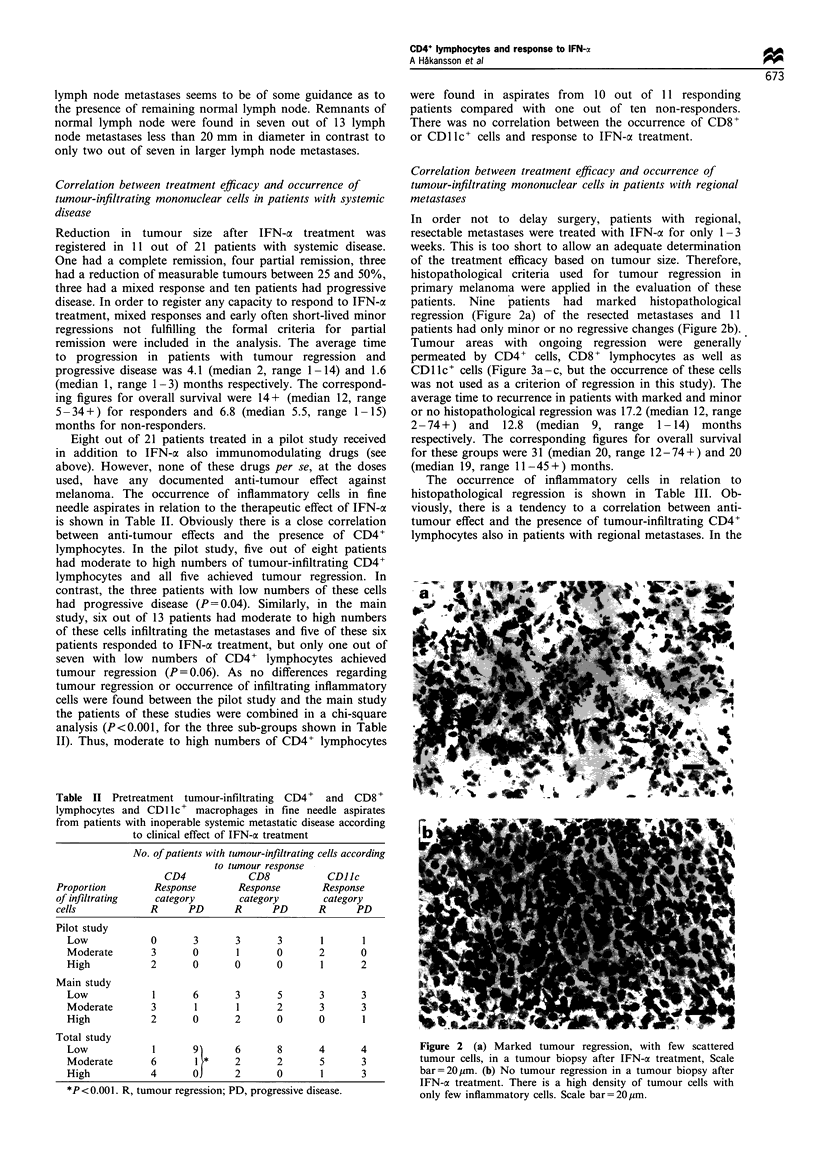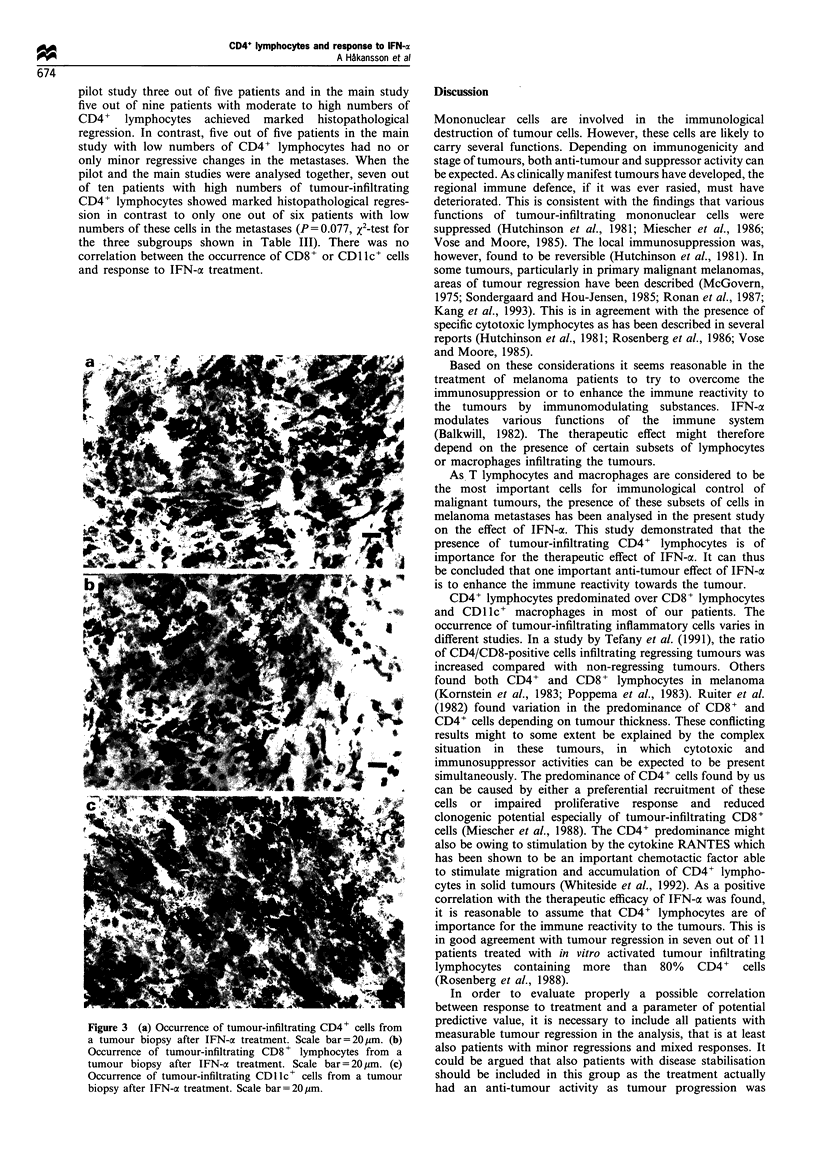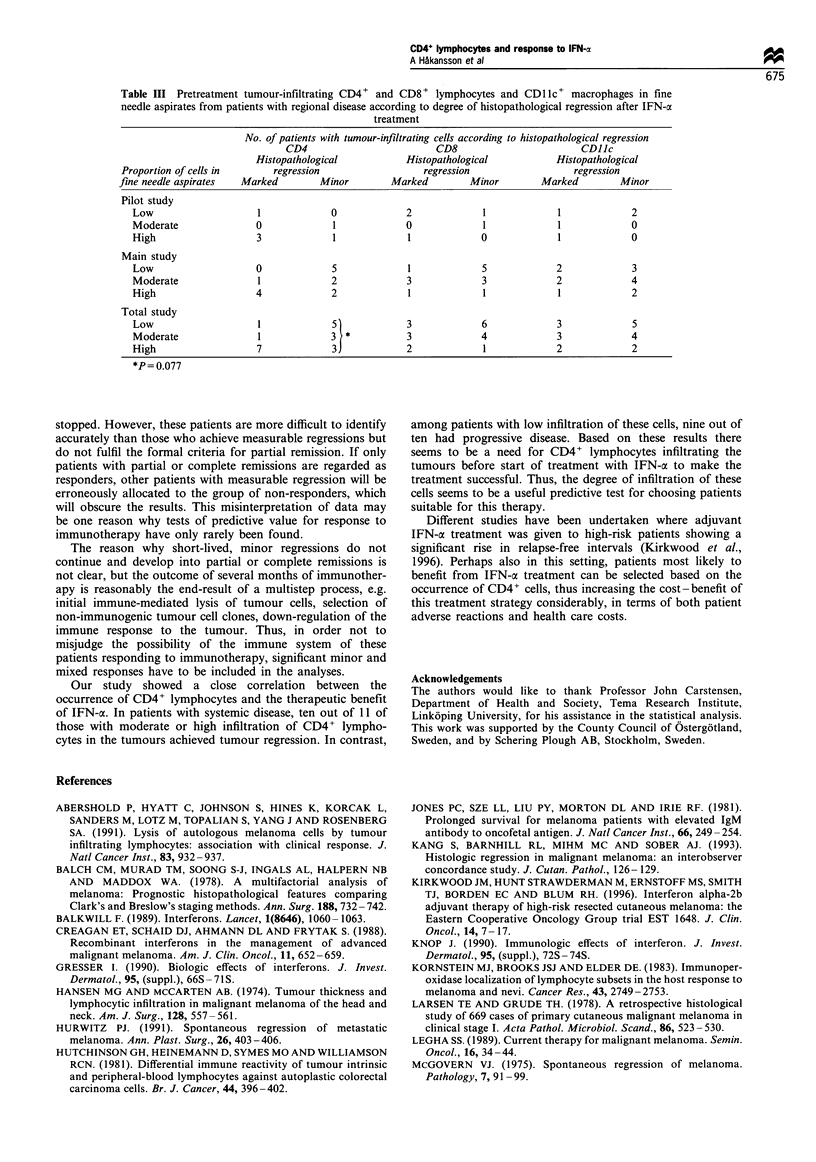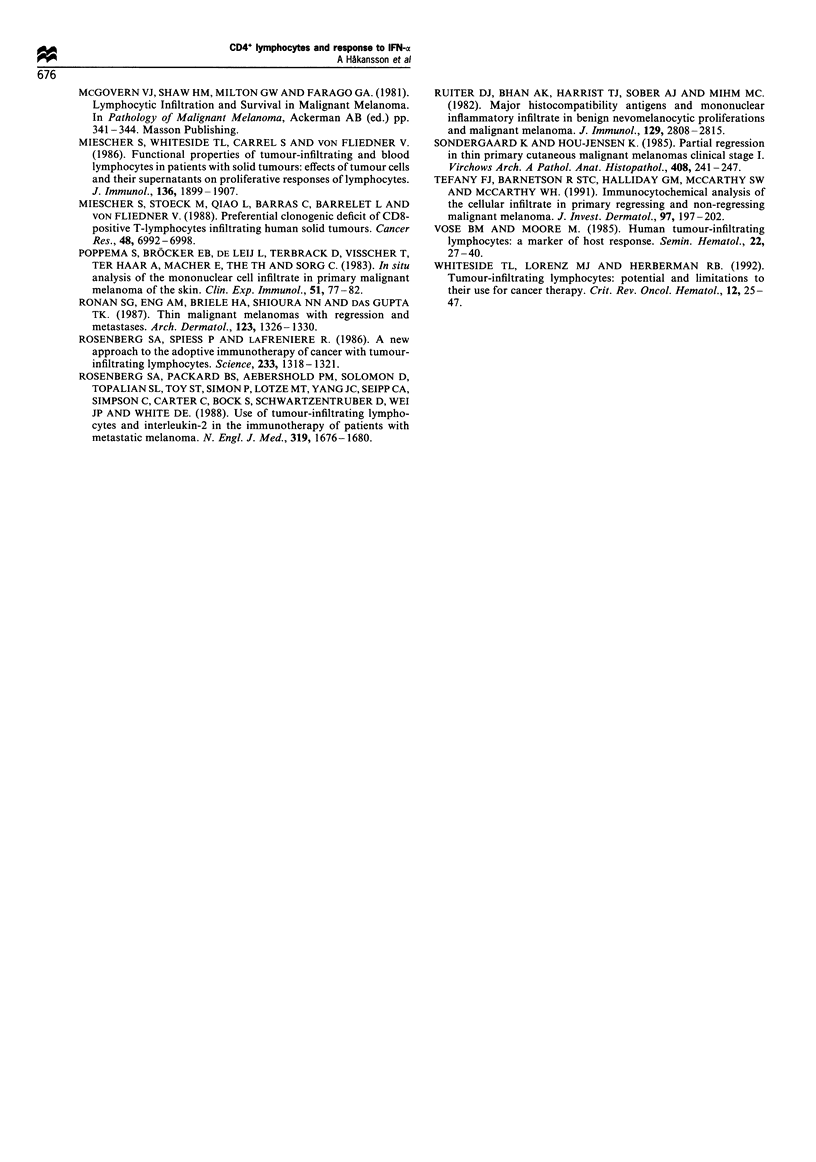# Tumour-infiltrating lymphocytes in metastatic malignant melanoma and response to interferon alpha treatment.

**DOI:** 10.1038/bjc.1996.420

**Published:** 1996-09

**Authors:** A. Håkansson, B. Gustafsson, L. Krysander, L. Håkansson

**Affiliations:** Department of Oncology, University Hospital, Linköping, Sweden.

## Abstract

**Images:**


British Journal of Cancer (1996) 74, 670–676
© 1996 Stockton Press All rights reserved 0007–0920/96 $12.00

### Tumour-infiltrating lymphocytes in metastatic malignant melanoma and response to interferon alpha treatment

A Håkansson[1], B Gustafsson[2], L Krysander[3] and L Håkansson[1]

*Departments of [1]Oncology, [2]Pathology and Cytology and [3]Hand and Plastic Surgery, University Hospital, S-581 85 Linköping, Sweden.*

**Summary** Interferon alpha (IFN-α) has a documented activity against malignant melanoma with a response rate of only approximately 20%. It would therefore be of considerable importance if patients likely to respond could be identified. The degree of mononuclear cell infiltration in primary tumours has been reported to correlate with a favourable prognosis. This investigation used monoclonal antibodies, anti-CD4, -CD8 and -CD11c, to identify subsets of tumour-infiltrating mononuclear cells in fine needle aspirates to study whether the presence of such cells correlates with the therapeutic effect of IFN-α. Twenty-one patients with systemic and 20 with regional metastatic malignant melanoma were studied before initiation of IFN-α treatment. A statistically significant correlation ($P < 0.001$) was found between the occurrence of $CD4^+$ lymphocytes in fine needle aspirates and the therapeutic benefit of IFN-α in patients with systemic disease. Ten out of 11 with moderate to high numbers of infiltrating $CD4^+$ lymphocytes achieved tumour regression. In contrast, among patients with low numbers of these cells in metastatic lesions, nine out of ten had progressive disease. Similar results were found in patients with regional disease.

**Keywords:** malignant melanoma; interferon alpha; tumour-infiltrating mononuclear cell; fine needle aspiration

Immune reactivity to malignant melanoma has been suggested by spontaneous regressions (Hurwitz, 1991), the occurrence of antibodies of prognostic significance against melanoma-associated antigens (Jones *et al.*, 1981) and specific cytotoxic lymphocytes (Abershold *et al.*, 1991). Conflicting results have, however, been reported on the prognostic significance of lymphocyte infiltration in primary malignant melanoma. Some studies found a significantly better prognosis if the primary lesions had a prominent infiltration of lymphocytes (Hansen and McCarten, 1974; Lasen and Grude, 1978), whereas others found no such correlation (Balch *et al.*, 1978). Three studies also found a relation between tumour thickness and lymphocyte infiltration (Balch *et al.*, 1978; Hansen and McCarten, 1974; Larsen and Grude, 1978). Tumour-infiltrating lymphocytes were further studied by McGovern *et al.* (1981), who found the infiltration at the base of the melanoma to be of prognostic significance in contrast to infiltration at the margins. McGovern *et al.* (1981) also found that the lymphocyte infiltration at the tumour base was reduced as tumour thickness increased. A reasonable strategy to treat malignant melanoma would therefore be to enhance the anti-tumour immune reactivity by immunomodulating substances, such as interferons and interleukin 2.

Interferon alpha (IFN-α) has a documented activity against metastatic malignant melanoma (Legha, 1989). Five to ten million units $m^{-2}$ IFN-α three times weekly, at the cost of reasonable side-effects, seems to be an optimal dose range (Legha, 1989). The treatment efficacy varies in different studies (Creagan *et al.*, 1988; Legha, 1989) and the overall response rate (CR+PR) to IFN-α alone is only about 20% (Creagan *et al.*, 1988; Legha, 1989). It would therefore be a considerable improvement if patients with a high probability of responding to this treatment could be identified using prognostic tests.

Besides having an anti-proliferative activity, the anti-tumour effect of IFN-α can be caused by the modulation of tumour cells, e.g. increased expression of cell surface proteins such as MHC I and tumour-associated antigens, which are of importance for the immunological control of tumours. In addition, IFN-α modulates several immune functions (Balkwill, 1982). There are still no firm data demonstrating which of the above-mentioned activities of IFN-α is the most important for the anti-tumour activity or whether they all contribute.

As IFN-α modulates the activity of various cells in the immune system (Knop, 1990; Gresser, 1990) the therapeutic effect might depend on the immune status of the patients when IFN-α therapy is initiated.

The aim of the present investigation was to study whether the presence of subsets of tumour-infiltrating mononuclear cells identified by immunocytochemical staining of fine needle aspirates of metastatic malignant melanoma correlates with the response to IFN-α.

### Materials and methods

### *Patient data*

This report describes 41 patients with metastatic malignant melanoma, 23 males and 18 females. Median age was 60 years (range 33–77 years) and Karnofsky performance status was 70 or more. Recurrences were cytologically verified by fine needle aspirates before start of treatment. Two groups of patients were studied, those with resectable regional disease and those with systemic disease. Twenty patients had metastases to regional lymph nodes, which in general were excised after 1 to 3 weeks of IFN-α treatment. Twenty-one patients had systemic disease with the following metastatic sites: two cutaneous, five subcutaneous, 16 lymph nodes, eight pulmonary, four bone and 16 visceral (nine liver, one ovarian, one pancreatic, one vulvar, one adrenal gland and three spleen metastases). The number of metastatic sites were one in six patients, two in seven patients, three in four patients, four in one patient and five or more in three patients. Except for one patient who had had chemotherapy, no patient had previously been treated except for surgical removal of primary lesions or metastases. Patients with symptoms of brain metastases were not included in this study.

### *Pretreatment investigations and treatment evaluation*

These included electrocardiograph, abdominal computed tomography, chest radiograph, bone scintigraphy and blood

Correspondence: A Håkansson
Received 18 September 1995; revised 8 March 1996; accepted 25 March 1996

samples for measurements of creatinine, bilirubin, alkaline phosphatase, alanine aminotransferase, lactate dehydrogenase, alpha amylase, haemoglobin, white blood cells and thrombocytes.

*Treatment schedule*

In a pilot study including 14 patients (six with regional disease and eight with systemic disease), IFN-α was given subcutaneously (s.c.) 3 days weekly at a dose of 10 million IU in combination with cyclophosphamide at a dose of 300 mg $m^{-2}$ administered as intravenous (i.v.) bolus every 3 weeks and seven of these patients were also treated with 50 mg of indomethacin three times daily. One patient was on cimetidine medication. This study was followed by the main study including 27 patients (14 with regional resectable disease and 13 with systemic disease). These patients were treated with IFN-α alone at the same dose and schedule as in the pilot study. In patients with regional resectable disease the treatment could only be given for 1–3 weeks in order not to delay surgery. In patients with distant metastases treatment continued until tumour progression. One treatment cycle included 3 weeks of treatment followed by 1 week without treatment. Tumour response was evaluated in patients with regional resectable disease after 1–3 weeks and in patients with systemic disease after one (clinically measurable disease) and three treatment cycles.

*Monoclonal antibodies*

*CD4 (Leu-3a, Becton-Dickinson)* The antigen is present on the helper/inducer T subset and in low density on monocytes and in the cytoplasm of monocytes and macrophages.

*CD8 (Leu-2a, Becton-Dickinson)* The antigen is present on cytotoxic/suppressor lymphocytes. The antigen is also expressed on some Leu-11$^+$ (CD16) cytotoxic natural killer (NK) cells, on a subpopulation of Leu-7$^+$ (HNK-1) cells (which do not have cytotoxic and NK activity), on some Leu-8$^+$ cells (which participate in suppression of B-cell function), and on Leu-15$^+$ (CD11b) cells, which are associated with suppressor function.

*CD11c (M5, Becton Dickinson)* The antigen is present on monocytes and in low density on granulocytes and large granular lymphocytes in peripheral blood. It is also expressed on macrophages in normal lymphoid tissue, on Kuppfer cells in liver and alveolar macrophages in lung tissue.

*Fine needle aspiration of metastases*

Usually seven to ten aspirates were taken from each tumour with a 0.6 mm hypodermic needle. The aspirate was smeared on a glass slide and was allowed to dry in air. At least two smears were then stained for conventional cytomorphology according to the May-Grunewald–Giemsa method. In cases without obvious melanin pigment in tumour cells, the diagnosis of melanoma was confirmed with immunostaining for vimentin and protein S-100. Morphological signs of degeneration or necrosis were registered. Aspirates were obtained from lymph node metastases in patients with regional disease and from five subcutaneous, two liver and 14 lymph node metastases in patients with systemic disease.

*Immunological staining of fine needle aspirates*

The slides were air dried and then fixed for 5 min in acetone. After drying, the slides were washed in phosphate-buffered saline (PBS), pH 7.6, and incubated with monoclonal antibodies against CD4, CD8 and CD11c (see above) for 30 min. Mouse IgG (Sigma, Stockholm, Sweden) was used as a negative control. After washing in PBS, the sections were incubated with rabbit anti-mouse immunoglobulin (Dakopatts Z 259) and incubated for 30 min, washed in PBS and incubated with the PAP mouse monoclonal antibody (Dakopatts, P 850) for 30 min. After washing in PBS, the slides were incubated in 50 ml PBS containing 40 mg diaminobenzidine, DAB (Sigma, Stockholm, Sweden) and 0.6 ml 3% hydrogen peroxide for 6 min and washed. The slides were counterstained in Mayer's haematoxylin for 15 min, washed and mounted in Glycergel (Dakopatts, Sweden). All incubations were performed in a moist chamber.

*Evaluation of mononuclear cells*

Because of the often heterogeneous distribution of infiltrating inflammatory cells, counting of cells per microscopic field was not performed. Instead, the overall occurrence of each subset of these cells, in relation to the number of tumour cells, was scored as low, moderate and high. CD4$^+$ or CD11c$^+$ cells scored as lymphocytes had small or medium-sized nuclei and sparse cytoplasm with distinct cell borders. In contrast, CD4$^+$ or CD11c$^+$ macrophages displayed large nuclei and abundant, generally faintly staining, cytoplasm. The proportion of lymphocytic *vs* non-lymphocytic CD4$^+$ or CD11c$^+$ cells was registered. CD8$^+$ cells always appeared as small or medium-sized lymphocytes.

*Preparation of tumour biopsies and immunological staining of tissue sections*

Biopsies from resected tumours were immediately snap frozen and stored at −70°C until further processed. Frozen tissue sections, 6–7 μm thick, were fixed in acetone for 10 min and then air dried. They were washed in Tris-buffered saline (TBS), pH 7.6, for 5 min, incubated with primary antibodies CD4, CD8 and CD11c (see above) for 30 min and then washed in TBS for 5 min. Mouse IgG (Sigma) was used as a negative control. The sections were then incubated with rabbit anti-mouse immunoglobulin (Dakopatts, Z 259) fo 30 min, washed in TBS and incubated with the APAAP mouse monoclonal antibody (Dakopatts D 651) for 30 min. After washing in TBS and incubating with the alkaline phosphatase substrate [naphthol AS-MX phosphate 2 mg (Sigma N4875), dimethylformamide 0.2 ml, 0.1 M Tris buffer, pH 8.2 9.8 ml, 1 M levamisole 50 μl (Sigma L-9756) and fast red TR salt 10 mg (Sigma F 1500)] for 20 min, the sections were washed again in TBS. They were then counterstained in Mayer's haematoxylin for 15 min and mounted in Glycergel (Dakopatts, Sweden). All incubations were performed in a moist chamber.

*Criteria of response in patients with systemic disease (according to WHO)*

*Complete response (CR)* Complete response was defined as disappearance of all known disease.

*Partial response (PR)* A PR was defined as decrease of at least 50% in the sum of the products of the largest perpendicular diameters of measurable lesions determined by two observations not less than 4 weeks apart. It is not necessary for all lesions to have regressed to qualify for partial response, but no lesion should have progressed and no new lesions should appear.

*Minor regressions* These did not fulfil the criteria for partial regression either because the reduction in the tumour size was 25–50% or because the duration of the response was too short.

*Mixed response* This was defined as measurable shrinkage of some lesion and simultaneous progressive disease in some other metastasis, or the appearance of new lesions.

*Stable disease (SD)* SD was considered to be present when a 25% decrease in total tumour size could not be established and a 25% increase in the size of one or more measurable lesions could not be demonstrated. In addition, there is no appearance of new lesions.

*Progressive disease (PD)* PD was defined as a 25% or more increase in the size of at least one measurable lesion or the appearance of a new lesion. As the objective of this study was to analyse a correlation between the occurrence of tumour-infiltrating inflammatory cells and the anti-tumour effect of IFN-α, significant tumour regression (more than 25%) in patients with minor regressions and mixed responses, not fulfilling the criteria for partial remission, were used in the following analysis.

*Evaluation of tumour regression in patients with regional metastases and criteria of histopathological tumour regression*

Patients with regional, resectable metastases were treated with IFN-α for 1–3 weeks before surgery. This is too short to allow an adequate determination of the treatment efficacy based on tumour size. Instead the occurrence of tumour regression was evaluated by histopathological examination of tumour biopsies. Based on the description of regressive changes in primary malignant melanoma in other studies (McGovern, 1975; Kang *et al.*, 1993; Sondergaard and Hou-Jensen, 1985; Ronan *et al.*, 1987), the following criteria of tumour regression were used in this study: (1) low and variable density of tumour cells, particularly variation in density within the same tumour nodule; (2) disorganisation of the architecture of the tumour with nests of remaining tumour cells surrounded by stromal tissue; (3) fibrosis. However, the inflammatory infiltrate was not used as a criterion of histopathological tumour regression in this study. The signs of regression vary from no signs to almost complete destruction with only a few tumour cells present. The degree of tumour regression was considered minor when regressive changes were estimated to be less than 25% (minor regression) and marked when such changes were estimated to be more than 25% (marked regression) of the section area.

*Statistical method*

For the final analyses the two studies were combined as no differences were found between them regarding response rates or histopathological regression or tumour-infiltrating inflammatory cells. The difference in distribution of inflammatory cells between patients with tumour response and progressive disease and between patients with and without histopathological tumour regression was analysed using the chi-square test. The pilot and main studies of patients with systemic disease were analysed using Fisher's exact test.

### Results

*Comparison of subsets of tumour-infiltrating mononuclear cells (MNCs) in fine needle aspirates in melanoma patients with regional and systemic metastases*

The number of tumour-infiltrating mononuclear cells in fine needle aspirates of melanoma metastases before initiation of IFN-α treatment (Figure 1) showed considerable individual variation (Table I). There was also a difference between metastases from patients with regional or systemic disease. High numbers of $CD4^+$ lymphocytes were found in metastases from 10 out of 20 patients with regional metastases but only in four out of 21 metastases from patients with systemic disease ($P<0.05$).

When $CD4^+$ cells were found in high numbers, more than 50% showed morphological characteristics of lymphocytes. In contrast, in tumours with low to moderate numbers of these cells, $CD4^+$ macrophages were more abundant than $CD4^+$ lymphocytes in four out of ten patients with regional disease and in two out of 17 patients with systemic disease.

There was no difference in the ratio of $CD4^+$ and $CD8^+$ cells between metastases from patients with regional or systemic disease. $CD4^+$ cells predominated over $CD8^+$ cells in seven out of 19 metastases from patients with regional disease and in six out of 20 patients with systemic disease. $CD8^+$ cells were more abundant than $CD4^+$ cells in two out of 19 and two out of 20 metastases from patients with regional and systemic disease respectively.

Metastases from patients with regional or systemic disease were infiltrated to the same extent by macrophages. High numbers of $CD11c^+$ macrophages were found in four out of 19 metastases from patients with regional and in four out of 20 tumours from patients with systemic disease.

*Comparison of tumour-infiltrating $CD4^+$ lymphocytes in fine needle aspirates and biopsies*

Even if IFN-α might influence the recruitment of $CD4^+$ cells to tumours, there was, in general, a close correlation between the occurrence of these cells in fine needle aspirates and in biopsies after IFN-α treatment in 15 out of 20 patients. Lower numbers of $CD4^+$ cells in tissue sections compared with aspirates were found in only three metastases, which might be explained by remnants of normal lymph node tissue. Alternatively a down-regulation might have occurred during IFN-α treatment. Biopsies from two patients showed an increase of $CD4^+$ cells compared with aspirates. The size of

**Figure 1** Occurrence of tumour-infiltrating $CD4^+$ lymphocytes in a fine needle aspirate from a metastasis before initiation of IFN-α treatment. Scale bar = 20 μm.

**Table I** Tumour-infiltrating $CD4^+$ and $CD8^+$ lymphocytes and $CD11c^+$ macrophages in fine needle aspirates from patients with regional and systemic disease before initiation of IFN-α treatment

| | *No. of patients with tumour-infiltrating cells before initiation of IFN-αtreatment* | | | | | |
|---|---|---|---|---|---|---|
| *Proportion of cells in* | *CD4* | | *CD8* | | *CD11c* | |
| *fine needle aspirates* | *Regional* | *Systemic* | *Regional* | *Systemic* | *Regional* | *Systemic* |
| Low | 6 | 10 | 9 | 14 | 8 | 8 |
| Moderate | 4 | 7 | 7 | 4 | 7 | 8 |
| High | 10 | 4 | 3 | 2 | 4 | 4 |

lymph node metastases seems to be of some guidance as to the presence of remaining normal lymph node. Remnants of normal lymph node were found in seven out of 13 lymph node metastases less than 20 mm in diameter in contrast to only two out of seven in larger lymph node metastases.

*Correlation between treatment efficacy and occurrence of tumour-infiltrating mononuclear cells in patients with systemic disease*

Reduction in tumour size after IFN-α treatment was registered in 11 out of 21 patients with systemic disease. One had a complete remission, four partial remission, three had a reduction of measurable tumours between 25 and 50%, three had a mixed response and ten patients had progressive disease. In order to register any capacity to respond to IFN-α treatment, mixed responses and early often short-lived minor regressions not fulfilling the formal criteria for partial remission were included in the analysis. The average time to progression in patients with tumour regression and progressive disease was 4.1 (median 2, range 1–14) and 1.6 (median 1, range 1–3) months respectively. The corresponding figures for overall survival were 14+ (median 12, range 5–34+) for responders and 6.8 (median 5.5, range 1–15) months for non-responders.

Eight out of 21 patients treated in a pilot study received in addition to IFN-α also immunomodulating drugs (see above). However, none of these drugs *per se*, at the doses used, have any documented anti-tumour effect against melanoma. The occurrence of inflammatory cells in fine needle aspirates in relation to the therapeutic effect of IFN-α is shown in Table II. Obviously there is a close correlation between anti-tumour effects and the presence of $CD4^+$ lymphocytes. In the pilot study, five out of eight patients had moderate to high numbers of tumour-infiltrating $CD4^+$ lymphocytes and all five achieved tumour regression. In contrast, the three patients with low numbers of these cells had progressive disease ($P = 0.04$). Similarly, in the main study, six out of 13 patients had moderate to high numbers of these cells infiltrating the metastases and five of these six patients responded to IFN-α treatment, but only one out of seven with low numbers of $CD4^+$ lymphocytes achieved tumour regression ($P = 0.06$). As no differences regarding tumour regression or occurrence of infiltrating inflammatory cells were found between the pilot study and the main study the patients of these studies were combined in a chi-square analysis ($P < 0.001$, for the three sub-groups shown in Table II). Thus, moderate to high numbers of $CD4^+$ lymphocytes

**Table II** Pretreatment tumour-infiltrating $CD4^+$ and $CD8^+$ lymphocytes and $CD11c^+$ macrophages in fine needle aspirates from patients with inoperable systemic metastatic disease according to clinical effect of IFN-α treatment

| | *No. of patients with tumour-infiltrating cells according to tumour response* | | | | | |
|---|---|---|---|---|---|---|
| | *CD4* | | *CD8* | | *CD11c* | |
| *Proportion of infiltrating cells* | *Response category* *R* | *PD* | *Response category* *R* | *PD* | *Response category* *R* | *PD* |
| Pilot study | | | | | | |
| Low | 0 | 3 | 3 | 3 | 1 | 1 |
| Moderate | 3 | 0 | 1 | 0 | 2 | 0 |
| High | 2 | 0 | 0 | 0 | 1 | 2 |
| Main study | | | | | | |
| Low | 1 | 6 | 3 | 5 | 3 | 3 |
| Moderate | 3 | 1 | 1 | 2 | 3 | 3 |
| High | 2 | 0 | 2 | 0 | 0 | 1 |
| Total study | | | | | | |
| Low | 1 | 9 | 6 | 8 | 4 | 4 |
| Moderate | 6 | 1 * | 2 | 2 | 5 | 3 |
| High | 4 | 0 | 2 | 0 | 1 | 3 |

*$P < 0.001$. R, tumour regression; PD, progressive disease.

were found in aspirates from 10 out of 11 responding patients compared with one out of ten non-responders. There was no correlation between the occurrence of $CD8^+$ or $CD11c^+$ cells and response to IFN-α treatment.

*Correlation between treatment efficacy and occurrence of tumour-infiltrating mononuclear cells in patients with regional metastases*

In order not to delay surgery, patients with regional, resectable metastases were treated with IFN-α for only 1–3 weeks. This is too short to allow an adequate determination of the treatment efficacy based on tumour size. Therefore, histopathological criteria used for tumour regression in primary melanoma were applied in the evaluation of these patients. Nine patients had marked histopathological regression (Figure 2a) of the resected metastases and 11 patients had only minor or no regressive changes (Figure 2b). Tumour areas with ongoing regression were generally permeated by $CD4^+$ cells, $CD8^+$ lymphocytes as well as $CD11c^+$ cells (Figure 3a–c, but the occurrence of these cells was not used as a criterion of regression in this study). The average time to recurrence in patients with marked and minor or no histopathological regression was 17.2 (median 12, range 2–74+) and 12.8 (median 9, range 1–14) months respectively. The corresponding figures for overall survival for these groups were 31 (median 20, range 12–74+) and 20 (median 19, range 11–45+) months.

The occurrence of inflammatory cells in relation to histopathological regression is shown in Table III. Obviously, there is a tendency to a correlation between anti-tumour effect and the presence of tumour-infiltrating $CD4^+$ lymphocytes also in patients with regional metastases. In the

**Figure 2** (**a**) Marked tumour regression, with few scattered tumour cells, in a tumour biopsy after IFN-α treatment, Scale bar = 20 μm. (**b**) No tumour regression in a tumour biopsy after IFN-α treatment. There is a high density of tumour cells with only few inflammatory cells. Scale bar = 20 μm.

pilot study three out of five patients and in the main study five out of nine patients with moderate to high numbers of $CD4^+$ lymphocytes achieved marked histopathological regression. In contrast, five out of five patients in the main study with low numbers of $CD4^+$ lymphocytes had no or only minor regressive changes in the metastases. When the pilot and the main studies were analysed together, seven out of ten patients with high numbers of tumour-infiltrating $CD4^+$ lymphocytes showed marked histopathological regression in contrast to only one out of six patients with low numbers of these cells in the metastases ($P=0.077$, $\chi^2$-test for the three subgroups shown in Table III). There was no correlation between the occurrence of $CD8^+$ or $CD11c^+$ cells and response to IFN-α treatment.

**Figure 3** (**a**) Occurrence of tumour-infiltrating $CD4^+$ cells from a tumour biopsy after IFN-α treatment. Scale bar = 20 μm. (**b**) Occurrence of tumour-infiltrating $CD8^+$ lymphocytes from a tumour biopsy after IFN-α treatment. Scale bar = 20 μm. (**c**) Occurrence of tumour-infiltrating $CD11c^+$ cells from a tumour biopsy after IFN-α treatment. Scale bar = 20 μm.

### Discussion

Mononuclear cells are involved in the immunological destruction of tumour cells. However, these cells are likely to carry several functions. Depending on immunogenicity and stage of tumours, both anti-tumour and suppressor activity can be expected. As clinically manifest tumours have developed, the regional immune defence, if it was ever rasied, must have deteriorated. This is consistent with the findings that various functions of tumour-infiltrating mononuclear cells were suppressed (Hutchinson *et al.*, 1981; Miescher *et al.*, 1986; Vose and Moore, 1985). The local immunosuppression was, however, found to be reversible (Hutchinson *et al.*, 1981). In some tumours, particularly in primary malignant melanomas, areas of tumour regression have been described (McGovern, 1975; Sondergaard and Hou-Jensen, 1985; Ronan *et al.*, 1987; Kang *et al.*, 1993). This is in agreement with the presence of specific cytotoxic lymphocytes as has been described in several reports (Hutchinson *et al.*, 1981; Rosenberg *et al.*, 1986; Vose and Moore, 1985).

Based on these considerations it seems reasonable in the treatment of melanoma patients to try to overcome the immunosuppression or to enhance the immune reactivity to the tumours by immunomodulating substances. IFN-α modulates various functions of the immune system (Balkwill, 1982). The therapeutic effect might therefore depend on the presence of certain subsets of lymphocytes or macrophages infiltrating the tumours.

As T lymphocytes and macrophages are considered to be the most important cells for immunological control of malignant tumours, the presence of these subsets of cells in melanoma metastases has been analysed in the present study on the effect of IFN-α. This study demonstrated that the presence of tumour-infiltrating $CD4^+$ lymphocytes is of importance for the therapeutic effect of IFN-α. It can thus be concluded that one important anti-tumour effect of IFN-α is to enhance the immune reactivity towards the tumour.

$CD4^+$ lymphocytes predominated over $CD8^+$ lymphocytes and $CD11c^+$ macrophages in most of our patients. The occurrence of tumour-infiltrating inflammatory cells varies in different studies. In a study by Tefany *et al.* (1991), the ratio of CD4/CD8-positive cells infiltrating regressing tumours was increased compared with non-regressing tumours. Others found both $CD4^+$ and $CD8^+$ lymphocytes in melanoma (Kornstein *et al.*, 1983; Poppema *et al.*, 1983). Ruiter *et al.* (1982) found variation in the predominance of $CD8^+$ and $CD4^+$ cells depending on tumour thickness. These conflicting results might to some extent be explained by the complex situation in these tumours, in which cytotoxic and immunosuppressor activities can be expected to be present simultaneously. The predominance of $CD4^+$ cells found by us can be caused by either a preferential recruitment of these cells or impaired proliferative response and reduced clonogenic potential especially of tumour-infiltrating $CD8^+$ cells (Miescher *et al.*, 1988). The $CD4^+$ predominance might also be owing to stimulation by the cytokine RANTES which has been shown to be an important chemotactic factor able to stimulate migration and accumulation of $CD4^+$ lymphocytes in solid tumours (Whiteside *et al.*, 1992). As a positive correlation with the therapeutic efficacy of IFN-α was found, it is reasonable to assume that $CD4^+$ lymphocytes are of importance for the immune reactivity to the tumours. This is in good agreement with tumour regression in seven out of 11 patients treated with *in vitro* activated tumour infiltrating lymphocytes containing more than 80% $CD4^+$ cells (Rosenberg *et al.*, 1988).

In order to evaluate properly a possible correlation between response to treatment and a parameter of potential predictive value, it is necessary to include all patients with measurable tumour regression in the analysis, that is at least also patients with minor regressions and mixed responses. It could be argued that also patients with disease stabilisation should be included in this group as the treatment actually had an anti-tumour activity as tumour progression was

Table III Pretreatment tumour-infiltrating CD4$^+$ and CD8$^+$ lymphocytes and CD11c$^+$ macrophages in fine needle aspirates from patients with regional disease according to degree of histopathological regression after IFN-α treatment

| Proportion of cells in fine needle aspirates | No. of patients with tumour-infiltrating cells according to histopathological regression: CD4 Histopathological regression Marked | CD4 Minor | CD8 Histopathological regression Marked | CD8 Minor | CD11c Histopathological regression Marked | CD11c Minor |
|---|---|---|---|---|---|---|
| Pilot study | | | | | | |
| Low | 1 | 0 | 2 | 1 | 1 | 2 |
| Moderate | 0 | 1 | 0 | 1 | 1 | 0 |
| High | 3 | 1 | 1 | 0 | 1 | 0 |
| Main study | | | | | | |
| Low | 0 | 5 | 1 | 5 | 2 | 3 |
| Moderate | 1 | 2 | 3 | 3 | 2 | 4 |
| High | 4 | 2 | 1 | 1 | 1 | 2 |
| Total study | | | | | | |
| Low | 1 | 5* | 3 | 6 | 3 | 5 |
| Moderate | 1 | 3* | 3 | 4 | 3 | 4 |
| High | 7 | 3* | 2 | 1 | 2 | 2 |

*$P=0.077$

stopped. However, these patients are more difficult to identify accurately than those who achieve measurable regressions but do not fulfil the formal criteria for partial remission. If only patients with partial or complete remissions are regarded as responders, other patients with measurable regression will be erroneously allocated to the group of non-responders, which will obscure the results. This misinterpretation of data may be one reason why tests of predictive value for response to immunotherapy have only rarely been found.

The reason why short-lived, minor regressions do not continue and develop into partial or complete remissions is not clear, but the outcome of several months of immunotherapy is reasonably the end-result of a multistep process, e.g. initial immune-mediated lysis of tumour cells, selection of non-immunogenic tumour cell clones, down-regulation of the immune response to the tumour. Thus, in order not to misjudge the possibility of the immune system of these patients responding to immunotherapy, significant minor and mixed responses have to be included in the analyses.

Our study showed a close correlation between the occurrence of CD4$^+$ lymphocytes and the therapeutic benefit of IFN-α. In patients with systemic disease, ten out of 11 of those with moderate or high infiltration of CD4$^+$ lymphocytes in the tumours achieved tumour regression. In contrast, among patients with low infiltration of these cells, nine out of ten had progressive disease. Based on these results there seems to be a need for CD4$^+$ lymphocytes infiltrating the tumours before start of treatment with IFN-α to make the treatment successful. Thus, the degree of infiltration of these cells seems to be a useful predictive test for choosing patients suitable for this therapy.

Different studies have been undertaken where adjuvant IFN-α treatment was given to high-risk patients showing a significant rise in relapse-free intervals (Kirkwood *et al.*, 1996). Perhaps also in this setting, patients most likely to benefit from IFN-α treatment can be selected based on the occurrence of CD4$^+$ cells, thus increasing the cost–benefit of this treatment strategy considerably, in terms of both patient adverse reactions and health care costs.

**Acknowledgements**
The authors would like to thank Professor John Carstensen, Department of Health and Society, Tema Research Institute, Linköping University, for his assistance in the statistical analysis. This work was supported by the County Council of Östergötland, Sweden, and by Schering Plough AB, Stockholm, Sweden.

### References

ABERSHOLD P, HYATT C, JOHNSON S, HINES K, KORCAK L, SANDERS M, LOTZ M, TOPALIAN S, YANG J AND ROSENBERG SA. (1991). Lysis of autologous melanoma cells by tumour infiltrating lymphocytes: association with clinical response. *J. Natl Cancer Inst.*, **83**, 932–937.

BALCH CM, MURAD TM, SOONG S-J, INGALS AL, HALPERN NB AND MADDOX WA. (1978). A multifactorial analysis of melanoma: Prognostic histopathological features comparing Clark's and Breslow's staging methods. *Ann. Surg.* **188**, 732–742.

BALKWILL F. (1989). Interferons. *Lancet*, **1(8646)**, 1060–1063.

CREAGAN ET, SCHAID DJ, AHMANN DL AND FRYTAK S. (1988). Recombinant interferons in the management of advanced malignant melanoma. *Am. J. Clin. Oncol.*, **11**, 652–659.

GRESSER I. (1990). Biologic effects of interferons. *J. Invest. Dermatol.*, **95**, (suppl.), 66S–71S.

HANSEN MG AND MCCARTEN AB. (1974). Tumour thickness and lymphocytic infiltration in malignant melanoma of the head and neck. *Am. J. Surg.*, **128**, 557–561.

HURWITZ PJ. (1991). Spontaneous regression of metastatic melanoma. *Ann. Plast. Surg.*, **26**, 403–406.

HUTCHINSON GH, HEINEMANN D, SYMES MO AND WILLIAMSON RCN. (1981). Differential immune reactivity of tumour intrinsic and peripheral-blood lymphocytes against autoplastic colorectal carcinoma cells. *Br. J. Cancer*, **44**, 396–402.

JONES PC, SZE LL, LIU PY, MORTON DL AND IRIE RF. (1981). Prolonged survival for melanoma patients with elevated IgM antibody to oncofetal antigen. *J. Natl Cancer Inst.*, **66**, 249–254.

KANG S, BARNHILL RL, MIHM MC AND SOBER AJ. (1993). Histologic regression in malignant melanoma: an interobserver concordance study. *J. Cutan. Pathol.*, 126–129.

KIRKWOOD JM, HUNT STRAWDERMAN M, ERNSTOFF MS, SMITH TJ, BORDEN EC AND BLUM RH. (1996). Interferon alpha-2b adjuvant therapy of high-risk resected cutaneous melanoma: the Eastern Cooperative Oncology Group trial EST 1648. *J. Clin. Oncol.*, **14**, 7–17.

KNOP J. (1990). Immunologic effects of interferon. *J. Invest. Dermatol.*, **95**, (suppl.), 72S–74S.

KORNSTEIN MJ, BROOKS JSJ AND ELDER DE. (1983). Immunoperoxidase localization of lymphocyte subsets in the host response to melanoma and nevi. *Cancer Res.*, **43**, 2749–2753.

LARSEN TE AND GRUDE TH. (1978). A retrospective histological study of 669 cases of primary cutaneous malignant melanoma in clinical stage I. *Acta Pathol. Microbiol. Scand.*, **86**, 523–530.

LEGHA SS. (1989). Current therapy for malignant melanoma. *Semin. Oncol.*, **16**, 34–44.

MCGOVERN VJ. (1975). Spontaneous regression of melanoma. *Pathology*, **7**, 91–99.

MCGOVERN VJ, SHAW HM, MILTON GW AND FARAGO GA. (1981). Lymphocytic Infiltration and Survival in Malignant Melanoma. In *Pathology of Malignant Melanoma*, Ackerman AB (ed.) pp. 341-344. Masson Publishing.

MIESCHER S, WHITESIDE TL, CARREL S AND VON FLIEDNER V. (1986). Functional properties of tumour-infiltrating and blood lymphocytes in patients with solid tumours: effects of tumour cells and their supernatants on proliferative responses of lymphocytes. *J. Immunol.*, **136,** 1899-1907.

MIESCHER S, STOECK M, QIAO L, BARRAS C, BARRELET L AND VON FLIEDNER V. (1988). Preferential clonogenic deficit of CD8-positive T-lymphocytes infiltrating human solid tumours. *Cancer Res.*, **48,** 6992-6998.

POPPEMA S, BRÖCKER EB, DE LEIJ L, TERBRACK D, VISSCHER T, TER HAAR A, MACHER E, THE TH AND SORG C. (1983). *In situ* analysis of the mononuclear cell infiltrate in primary malignant melanoma of the skin. *Clin. Exp. Immunol.*, **51,** 77-82.

RONAN SG, ENG AM, BRIELE HA, SHIOURA NN AND DAS GUPTA TK. (1987). Thin malignant melanomas with regression and metastases. *Arch. Dermatol.*, **123,** 1326-1330.

ROSENBERG SA, SPIESS P AND LAFRENIERE R. (1986). A new approach to the adoptive immunotherapy of cancer with tumour-infiltrating lymphocytes. *Science*, **233,** 1318-1321.

ROSENBERG SA, PACKARD BS, AEBERSHOLD PM, SOLOMON D, TOPALIAN SL, TOY ST, SIMON P, LOTZE MT, YANG JC, SEIPP CA, SIMPSON C, CARTER C, BOCK S, SCHWARTZENTRUBER D, WEI JP AND WHITE DE. (1988). Use of tumour-infiltrating lymphocytes and interleukin-2 in the immunotherapy of patients with metastatic melanoma. *N. Engl. J. Med.*, **319,** 1676-1680.

RUITER DJ, BHAN AK, HARRIST TJ, SOBER AJ AND MIHM MC. (1982). Major histocompatibility antigens and mononuclear inflammatory infiltrate in benign nevomelanocytic proliferations and malignant melanoma. *J. Immunol.*, **129,** 2808-2815.

SONDERGAARD K AND HOU-JENSEN K. (1985). Partial regression in thin primary cutaneous malignant melanomas clinical stage I. *Virchows Arch. A Pathol. Anat. Histopathol.*, **408,** 241-247.

TEFANY FJ, BARNETSON R STC, HALLIDAY GM, MCCARTHY SW AND MCCARTHY WH. (1991). Immunocytochemical analysis of the cellular infiltrate in primary regressing and non-regressing malignant melanoma. *J. Invest. Dermatol.*, **97,** 197-202.

VOSE BM AND MOORE M. (1985). Human tumour-infiltrating lymphocytes: a marker of host response. *Semin. Hematol.*, **22,** 27-40.

WHITESIDE TL, LORENZ MJ AND HERBERMAN RB. (1992). Tumour-infiltrating lymphocytes: potential and limitations to their use for cancer therapy. *Crit. Rev. Oncol. Hematol.*, **12,** 25-47.